# Progressive hemifacial atrophy in a Chinese patient: A case report

**DOI:** 10.1097/MD.0000000000031872

**Published:** 2022-11-18

**Authors:** Rongrong Li, Haiyan Yu, Xizi Wang, Weifei Wang, Lili Yan, Fangjie Guo, Conghui Tian, Xiaoling Yuan, Min Zhao, Juan Zheng, Mingliang Gu, Xiaodong Jia, Dianrong Gong

**Affiliations:** a Department of Joint Laboratory for Translational Medicine Research, Liaocheng People’s Hospital, Liaocheng, China; b Department of Neurology, Liaocheng People’s Hospital, Liaocheng, China; c Beijing Institute of Genomics, Chinese Academy of Sciences and Key Laboratory of Genome Science and Information, Chinese Academy of Sciences, Beijing, China.

**Keywords:** *ARHGAP4* mutation, *CFAP47* mutation, etiology, *p*rogressive hemifacial atrophy, *w*hole-exome sequencing

## Abstract

Progressive hemifacial atrophy (PHA) is a rare and progressive condition of unknown etiology that is characterized by chronic progressive atrophy of the skin, subcutaneous tissue, muscle, and bone on 1 side of the face. However, its precise pathogenesis remains poorly understood.

**Case presentation:**

Here, we report a case of PHA, which manifested as left-sided facial atrophy. Whole-exome sequencing of peripheral blood samples from the patient and his parents, together with bioinformatics analyses, led to the identification of mutations in *ARHGAP4* and *CFAP47*.

**Conclusion:**

This report is the first to describe *ARHGAP4* and *CFAP47* mutations in a patient with PHA. These mutations may be related to the occurrence of hemifacial atrophy, although further studies are needed to clarify the role of *ARHGAP4* and *CFAP47* in the context of PHA pathogenesis.

## 1. Introduction

Progressive hemifacial atrophy (PHA) is a rare disorder wherein patients experience the gradual, progressive atrophy of the skin, subcutaneous fat, muscle, and bone on 1 side of the face.^[1,2]^ First reported in 1825 by Parry and codified as Parry-Romberg syndrome in 1846 by Romberg,^[3,4]^ the current name of this condition (PHA) was first proposed in 1871 by Enlenburg.^[5,6]^ The disease is characterized by onset in the first or second decade, and the number of female patients was approximately twice as much as that of male.^[7]^ Current published estimates suggest that PHA affects approximately 1 in 700,000 individuals.^[4,7]^ The clinical diagnosis of affected patients is mainly based on the identification of symptoms of PHA. PHA can have a serious adverse impact on the structure, function, and aesthetic characteristics of the oral and jaw systems in affected patients, negatively impacting their physical and mental health. There is thus a clear need for further study of the pathogenesis of PHA in order to guide the treatment of this condition. At present, although various hypotheses have been put forth, the cause of this disease remains unclear and no effective treatments are available. The leading theory is that PHA occurs due to a genetic alteration during the first stage of the embryogenic development of the central nervous system,^[8]^ but the specific affected gene remains unclear. Herein, we describe the case of a PHA patient for whom whole-exome sequencing (WES) was performed in an effort to detect potentially pathogenic gene mutations.

## 2. Case presentation

The Ethics Committee of Liaocheng People’s Hospital approved this case report, and the patient provided written informed consent for the publication of this report and the accompanying images.

**Figure 1.**
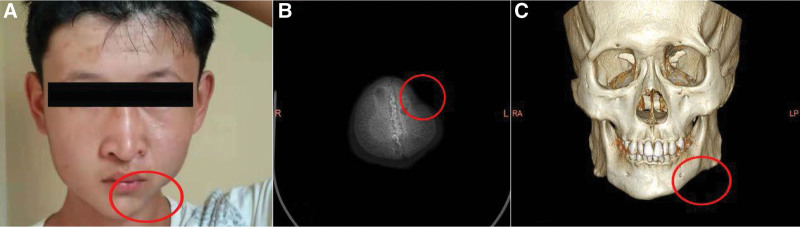
The clinical manifestations of PHA. (A) Frontal view of the patient. (B) A plain CT scan of the patient’s head revealed a narrow bilateral frontal cranial cavity and a significant left cranial depression. (C) A CT scan of the patient’s upper and lower jaw indicated that the left mandible was more concave relative to the contralateral side. PHA = progressive hemifacial atrophy.

The patient, a boy who was born on October 19, 2000, and he was 14 years old at the time of presentation, was diagnosed with PHA on April 13, 2015 at our hospital owing to a 1-year history of left-sided facial atrophy. At this time the patient’s frontal face and CT scan were obtained, and he has been followed up clinically since then. The patient had a complete inpatient record and diagnostic certificate following the first diagnosis in 2015. The patient’s parents exhibited no relevant family history of similar disease and were not affected by PHA. The patient presented with normal blood pressure and blood sugar levels, normal cognitive function, fluent speech, a positive attitude, and was able to freely move his eyes in all directions. He had no history of trauma, infection, or poisoning prior to the development of this disease, which exhibited an insidious onset. As shown in Figure 1A, the patient presented with bilateral asymmetry, with his face being smaller on the left side relative to the right side, together with atrophy of the left zygoma, cheek, mandible, and lip. There is a ~5 cm × 6 cm depression on the top of the left skull with a maximal depth of 0.5 cm. No abnormalities in speech, chewing, or swallowing were evident, nor did the patient experience any facial paresthesia. Plain CT scans of the skull revealed that the patient’s cranial cavity was narrow in the bilateral frontal region and that the left cranial depression was markedly sunken (Fig. 1B). CT scans of the upper and lower jaw indicated that the left mandible was more concave relative to the contralateral mandible (Fig. 1C).

### 2.1. Whole exome sequencing

In order to assess whether this patient harbored any pathogenic genetic mutations, the neurology department of our hospital collected 5 mL samples of peripheral blood from the patient and his healthy parents in 2022 for whole-exome sequencing, which was performed as follows:

Samples of genomic DNA (200 ng) were prepared from 5 mL peripheral blood samples collected from the patient and his healthy parents using a QIAamp DNA blood MIDI Kit (Qiagen, Germany). A Nanodrop instrument (Thermo Fisher Scientific, USA) was used to ensure the quality of the prepared DNA (OD 260/280 = 1.8–2.0), while the gDNA concentration was measured with a Qubit dsDNA Assay. These 200 ng gDNA samples were then diluted to a final volume of 50 µL using 1X Low TE Buffer, and DNA shearing was performed with a Covaris S220 instrument with a target DNA fragment size of 150 to 200 bp. An Agilent 2100 instrument was then used for quality control analyses of the fragmented DNA.

An Agilent Sureselect DNA Targeting Sequence Capture Kit was utilized for library preparation based on provided directions. Briefly, end-repair was first conducted for the fragmented DNA, with an A being added to the 3’ end of each fragment, and with the gap being connected using appropriate adapters. AMPure XP beads were used for purification after each step. Polymerase chain reaction was performed with an amplification volume of 50 µL, and the product was purified with AMPure XP beads. Amplified DNA was hybridized and placed at 65°C for 16 to 24 hours. After hybridization, stranded penicillin magnetic beads were applied for probe capture and Polymerase chain reaction amplification in a 50 µL volume. AMPure XP beads were again used for purification, and an Agilent 2100 instrument was used for quality control. The resultant fragment size for the final library was about 250 to 350 bp. Paired-end sequencing (PE75) was then performed with a Nextseq CN500 instrument (Illumina).

Sequencing data were analyzed by initially using Trimmatomatic to remove the original sequencing adapters and low-quality sequences. Filtered sequences were aligned via a BWA approach to the Thousands Genome Reference Sequence (GRCh38), and Samtools was used to convert the output data to a BAM file. Picard was used for sequence deduplication, and the GATK software was used to analyze and filter single nucleotide variation and insertion-deletion mutations. Called variants were mapped to the dbSNP database and annotated with ANNOVAR, and MAF values were filtered using the Exac database.

The resultant sequencing data were then analyzed. First, non-synonymous mutations in exons were screened, with those exhibiting a MAF < 0.01 in East Asian populations being retained. Heterozygous mutations that were present in the patient but absent in his parents were then retained, as were homozygous mutations that were present in the patient but heterozygous or absent in his parents. Through this approach, 2 mutation sites were identified (Table 1).

**Table 1:** Whole-exome sequencing results identifying potential pathogenic mutations associated with PHA incidence.

SNP	Gene	POS	REF	ALT	Case	Mother	Father
rs201123211	*ARHGAP4*	Xq28	C	T	1/1	0/1	0/0
rs142417700	*CFAP47*	Xp21.1	G	A	1/1	0/1	0/0

By annotating low-frequency mutation loci, we identified mutations in the *ARHGAP4* and *CFAP47* genes in this PHA patient. The rs201123211 mutation in *ARHGAP4* resulted in a change in the 891^st^ amino acid of this protein from glycine to aspartic acid. The rs142417700 mutation in *CFAP47* resulted in a change in the 870^th^ amino acid of this protein from arginine to glutamine.

### 2.2. Sanger sequencing validation

**Figure 2.**
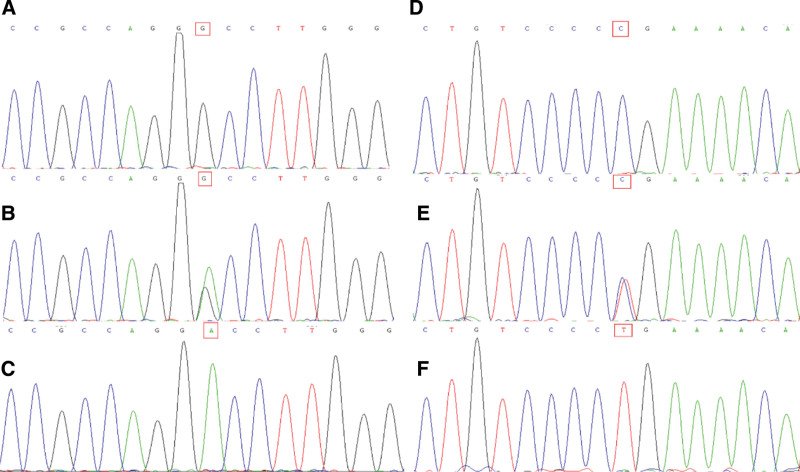
Identification of *ARHGAP4* and *CFAP47* mutations in a PHA patient. A-C) A portion of the *ARHGAP4* sequence from (A) the patient’s father, (B) the patient’s mother, and (C) the patient, with red frame being used to mark the mutation that was present in the patient but absent in both parents. (D–F) A portion of the *CFAP47* sequence from (D) the patient’s father, (E) the patient’s mother, and (F) the patient, with red frame being used to mark the mutation that was present in the patient but absent in both parents.

The reliability of these sequencing results was confirmed via Sanger sequencing. The results indicated that the patient did harbor mutations in *ARHGAP4* and *CFAP47* (Fig. 2).

## 3. Discussion

PHA is a rare disease that is primarily characterized by progressive asymmetric hemifacial atrophy. The pathogenesis of PHA remains unclear, and the neurologic disturbance of fat metabolism, which resulting from a trophic malformation of the cervical sympathetic nervous system has been proposed as a primary cause.^[9]^ Some researchers speculate that affected patients may exhibit defects in the genes controlling sympathetic nerves, resulting in cervical sympathetic nerve damage during development and consequent facial tissue neurodystrophy.^[2]^ While several hypotheses have been put forth regarding the etiology of PHA, none can fully explain the characteristics of this disease at present. Few studies exploring potential genes associated with the pathogenesis of this disease via WES have been published to date. In 2019, Liu et al first reported the case of a Chinese patient with PHA eye phenotypes harboring mutations in the *CRB1* gene,^[10]^ but other mutations have not been described to date. This study is the first report of *ARHGAP4* and *CFAP47* mutations in a patient with PHA.

*ARHGAP4* is a ~200 kb gene located on chromosome Xq28 (X: 153, 172, 829–153, 191, 776) (NCBI) that was identified by Tribioli in 1996, when he produced a transcriptional map of the Xq28 region. It encodes a 3.5 kb mRNA and a 946 amino acid protein.^[11]^ The *ARHGAP4* mRNA is highly expressed during development and in the adult central nervous system.^[12]^ *ARHGAP4* has been identified as a causative factor linked with a new phenotype characterized by nephrogenic diabetes insipidus, psychomotor delays, and short stature (Online Mendelian Inheritance in Man). Analyses of the genetic defects causing X-linked nephrogenic diabetes insipidus and intellectual disability in 2 dizygotic twin brothers revealed continuous deletions extending to intron 7 of the *ARHGAP4* gene, with dysmorphic facial features having been observed in 1 of the twins.^[13]^ The expression of *TRIM25*, which interacts with *ARHGAP4*, can induce adipocyte differentiation during adipogenesis. *ARHGAP4* encodes a protein with a proline-rich structure amenable to cellular scaffold formation and the maintenance of normal intracellular vesicle transport and endocytosis.^[14,15]^ Mutations in *ARHGAP4* result in the inhibition of its binding with proline structural proteins, potentially interfering with its ability to participate in cytoskeleton formation. *ARHGAP4* is expressed at significant levels in multiple specific tissues in both the developing and adult nervous systems, suggesting it to play an important role in this setting. This evidence thus suggests *ARHGAP4* mutations may be associated with PHA.

*CFAP47* (formerly known as CXorf22) is located on the human chromosome Xp21.1 and the gene encodes a cilia- and flagella-associated protein. The *CFAP47* gene has recently been linked to rare cases of asthenoazoospermia and male infertility.^[16]^ When the secondary structure of the *CFAP47* protein was evaluated using Interpro, amino acids 132-1303 were predicted to comprise an immunoglobulin-like (Ig-like) folding domain, while amino acids 1746 to 1869 were predicted to comprise a calponin homology domain. Ig-like domains primarily serve to interact with other Ig-like domains. Some studies have suggested a link between *CFAP47* and Sweeney-Cox syndrome,^[17]^ which is also characterized by significant facial dysplasia, including eyelid and facial bone defects and cleft lip formation. Human phenotypes related to Sweeney-Cox syndrome involve the facial nerves of the central nervous system.^[18]^ We thus further posit that *CFAP47* may act in a particular region of the nervous system such that *CFAP47* mutations can cause abnormal facial development, thereby promoting hemifacial atrophy.

The parents of the patient described in this report did not exhibit any PHA-related symptoms, nor did they suffer from any neurological diseases. The patient exhibited typical symptoms of PHA and had no history of congenital facial abnormalities or trauma, and these were thus excluded as potential causes. WES results from this patient identified 2 mutations in *ARHGAP4* and *CFAP47* that have the potential to be linked to the occurrence of PHA. As no specific or robust link between the *ARHGAP4*/*CFAP47* genes and PHA has been identified to date, further research will be needed to understand whether they contribute to the incidence of this degenerative disease. Overall, our study highlights the importance of conducting in-depth analyses of the pathogenesis of PHA, providing a potential direction for future diagnostic and research efforts pertaining to this disease.

## Acknowledgments

We thank the family for participating in this study and contributing to the literature.

## Authors contributions

**Conceptualization:** Rongrong Li, Dianrong Gong.

**Data curation:** Haiyan Yu, Xiaodong Jia, Mingliang Gu.

**Formal analysis:** Xizi Wang, Xiaodong Jia.

**Investigation:** Weifei Wang.

**Methodology:** Lili Yan, Fangjie Guo, Conghui Tian.

**Project administration:** Rongrong Li.

**Resources:** Dianrong Gong, Xiaodong Jia.

**Software:** Xizi Wang, Juan Zheng.

**Supervision:** Xiaoling Yuan, Min Zhao.

**Validation:** Rongrong Li. Xizi Wang.

**Visualization:** Rongrong Li, Haiyan Yu.

**Writing – original draft:** Rongrong Li, Dianrong Gong.

**Writing – review & editing:** Rongrong Li, Haiyan Yu Xiaodong Jia.
